# Exemplary Self-Discipline, Leniency towards Others: Competitive Contexts Amplify the “Black Sheep Effect” in Restoring Ingroup Trust

**DOI:** 10.3390/bs14070519

**Published:** 2024-06-21

**Authors:** Ningmeng Cao, Runrun Miao, Binghai Sun, Zirong Ren, Guoan Yue

**Affiliations:** 1 Zhejiang Normal University, Jinhua 321001, China; 2 School of Psychology, Zhejiang Normal University, Jinhua 321001, China; 3 College of Education, Zhejiang Normal University, Jinhua 321001, China

**Keywords:** group identity, trust repair, black sheep effect, form of intergroup interaction

## Abstract

Intergroup interaction, a pivotal aspect of social interaction, encompasses both cooperation and competition. Group identity significantly impacts individual behaviors and decision-making processes. This influence manifests in two contrasting ways when addressing rule-breaking by interaction partners: in-group favoritism, where individuals are more lenient towards infractions committed by in-group members, and the black sheep effect, where in-group members are penalized for their rule-breaking. Although trust is crucial in intergroup interactions, the precise impact of group identity on trust restoration and the potential moderating role of intergroup interaction types remain to be elucidated. This study presents two experiments designed to explore these dynamics. In Study 1, the manipulation of group identity through a point estimation task was utilized to evaluate its impact on intergroup trust restoration via a series of repeated trust games. Study 2 aimed to explore the moderating role of intergroup interaction on intergroup trust restoration by contrasting cooperation and competition situations. The results uncovered a “black sheep effect”, where participants demonstrated a greater propensity for trust restoration with out-group members than with in-group members. This effect, however, was only evident in competitive contexts. Conversely, in cooperative contexts, the individual’s trust in the in-group and out-group members is effectively repaired. These findings contribute to a deeper comprehension of trust dynamics in intergroup interactions, promoting trust establishment and repair between diverse groups, thereby boosting team collaboration efficiency and mitigating conflicts.

## 1. Introduction

The adage “birds of a feather flock together” underscores the social nature of group-living species, where individuals engage in cooperative and interactive exchanges with diverse communities throughout their social lives. Trust, the cornerstone of interpersonal interactions, denotes how individuals are prepared to place their faith and depend on another. It is not only pivotal in personal communication but also significantly influences intergroup relations. Categorized by the trustee’s social group, this trust can be classified as either an in-group or out-group trust, depending on the trustee’s affiliation [1]. In today’s rapidly globalizing context, characterized by accelerated communication, diplomacy, economic integration, and cultural exchange, the importance of cooperation among individuals from diverse backgrounds has reached unprecedented heights. Given the limited reliance on personal attributes, group identity often assumes a central role in shaping trust in these collaborative interactions.

Social identity theory (SIT) posits that individuals categorize themselves to differentiate between in-groups and out-groups and internalize the characteristics of their group as part of their self-concept. When individuals are in intergroup situations, their identification with their own group often leads to in-group favoritism or out-group discrimination, while out-group members experience less support and negative assessments [2,3,4,5]. This phenomenon, known as “in-group favoritism”, can arise naturally or through assigned group labels. Numerous studies have demonstrated that in-group favoritism fosters positive outcomes for group members, contributing to cohesion, collective identity, and cooperation [6,7,8,9]. Similarly, in-group favoritism influences trust behavior, with individuals more likely to trust their in-group members [10,11,12]. For instance, Mcleish and Oxoby (2011) [13] observed that individuals allocate more resources and express greater trust towards in-group members in trust games and ultimatum games.

Some studies [14,15,16] have demonstrated that individuals often exhibit in-group favoritism while simultaneously enacting more severe punishments on in-group members who transgress moral norms. This paradoxical behavior, characterized by heightened penalties for in-group transgressions, is termed the “black sheep effect”, contrasting with the typical bias towards in-group members. A study by Wu and Gao (2018) [17] indicated that girls aged 5 to 6 were more inclined to penalize in-group rule violators than out-group ones. Similarly, Yudkin et al. (2020) [18] found that a larger proportion of children opted to punish in-group wrongdoers compared to out-group violators (43% vs. 17%) upon witnessing moral infractions. Furthermore, the black sheep effect is also evident in the context of trust restoration. Fulmer and Gelfand (2015) [19] observed that individuals with collectivist tendencies experienced a slower and more challenging process when restoring trust with in-group members. In line with this, Zhou (2018) [20] noted that university students were more rapid in their trust recovery with out-group members as opposed to in-group peers.

Both in-group favoritism and the black sheep effect can influence the dynamics of individual trust. Specifically, the process of trust repair following a violation—particularly when it involves perceived distrust from both in-group and out-group members—requires a delicate interplay between these two opposing biases. Current scholarly work, such as that by Zheng, Qi, Wang, Zhao, Wang, & Gao (2020) [21], predominantly utilizes frameworks like mere preference theory (MPT) and normal focus theory (NFT) to elucidate the mechanisms underlying these group-based biases. The MPT suggests that once individuals perceive a strong affiliation with a group, they are inclined to view both the group and its members positively, which can result in a propensity to indulge, forgive, and conceal the group members’ shortcomings [22,23]. Consequently, negative actions by in-group members may be mitigated by the positive regard stemming from group identification, thereby potentially diminishing both the incidence and intensity of punitive responses [23]. Conversely, the NFT proposes that in-group transgressions pose a threat to the foundational values of the group, potentially jeopardizing group identity. Consequently, individuals may respond with heightened severity when in-group members engage in unfair behavior [23].

Cooperation and competition represent prevalent forms of interpersonal interaction, with the establishment and persistence of groups naturally engendering patterns of cooperation, competition, and conflict, both internally and across group boundaries. Studies have demonstrated that intragroup competition can suppress cooperation, diminish interpersonal appeal and trust, and provoke in-group conflict, thereby eroding in-group preference [24,25,26]. Conversely, other research indicates that intergroup competition, when endorsed by both in-group and out-group members, can diminish the prosocial behavior of children aged 5–10 towards the out-group, prompting them to allocate a larger share of resources to their in-group [27,28,29]. This suggests that intergroup competition can exacerbate in-group favoritism. In cooperative contexts, individuals often perceive their partners as closer and more intimate, exhibiting greater favoritism and trust towards them [30,31,32]. As a result, group bias may be less pronounced. This suggests that the form of intergroup interaction, whether cooperative or competitive, may influence the dynamics of trust repair, modulating the balance between in-group preference and the black sheep effect.

In summary, this study endeavors to explore the influence of group identity on intergroup trust repair, with a particular focus on the moderating role of various intergroup interaction types. This study aims to uncover the delicate equilibrium between individual inclinations towards in-group favoritism and the distancing from perceived “black sheep” during the trust repair process. To achieve this, Study 1 employs the minimal group paradigm to manipulate participants’ group identities and employs a repeated trust game to assess the impact of diverse group identities on intergroup trust restoration. Subsequently, Study 2 introduces cooperative and competitive situations to delve deeper into how intergroup interaction dynamics modulate trust repair dynamics, specifically towards both in-group and out-group members.

## 2. Study 1

Study 1 utilized a point-estimation task to manipulate the identities of the ingroup and outgroup for both trust parties. It also employed a repeated trust game to establish a trust process, examining the effects of the ingroup and outgroup identities of trust violators on intergroup trust repair. We hypothesize that an individual’s in-group and out-group identities influence the effectiveness of group trust repair. Specifically, individuals tend to repair trust with out-groups more effectively than within-groups, a phenomenon known as the “black sheep effect.”

### 2.1. Method

#### 2.1.1. Participant

A total of 148 nonpsychology undergraduate students, consisting of 58 males and 90 females, with an average age of 20.80 ± 2.17 years, were recruited for the study from a priori power analyses (α = 0.05, *power* = 0.80). Participants were randomly distributed into either the in-group (*n* = 76) or out-group (*n* = 72) condition. Eligibility criteria included normal or corrected vision, right-handedness, and voluntary participation. Post-experiment, participants received compensation proportional to their performance.

#### 2.1.2. Measures

**Trust game**. This study employed the well-established multiround trust game, as described by Berg, Dickhaut, and McCabe (1995) [33], involving two roles: investors and investees, or trustors and trustees. Each participant, functioning as a trustor, engaged in iterative investment rounds with a computer-simulated trustee, initially possessing 100 tokens per round. Participants were to decide whether to invest X tokens (0 ≤ X ≤ 100), with noninvestment resulting in a 100-token split. If they chose to invest, their amount would triple, and the trustee would decide to return half (3X/2) or retain the full 3X. Regardless of the trustee’s choice, the round concluded, and both parties received their tokens. The investment decision symbolized trust. This study manipulated trustee behavior by alternating between return scenarios in rounds 1–4, no returns in rounds 5 and 6, and returns in round 7, following an apology at the end of round 6.

**Trust propensity.** This study employed the Trust Propensity Scale, as previously utilized by Frazier, Johnson, and Fainshmidt (2013) [34], comprising four items, including the statement, “I usually trust people unless they provide a reason not to trust them”. The scale employs a Likert five-point scale ranging from 1 (strongly disagree) to 5 (strongly agree), with a higher total score reflecting a stronger inclination towards trust. The scale demonstrated strong internal consistency in this study, with a Cronbach’s alpha of 0.89.

**Trait forgiveness.** The Trait Forgiveness Scale (TFS) was employed to assess an individual’s propensity to forgive interpersonal transgressions [35]. This Likert-type scale encompasses 10 items, including 5 that are reverse-scored. The scale employs a Likert five-point scale ranging from 1 (strongly disagree) to 5 (strongly agree). The total score, calculated by summing the responses, reflects a higher level of forgiveness tendency. In this study, the scale exhibited an internal consistency of α = 0.75.

#### 2.1.3. Procedure

Upon arrival at the laboratory, participants first completed an informed consent form and provided essential demographic details. Following this, the experimenter then outlined the experimental protocol, ensuring participants’ comprehension before proceeding to the main task. The experimental procedure comprised two stages. In the first part, participants engaged in a point estimation task, specifically the group classification task as described by Durrheim et al. (2016) [36]. Each dot plot appeared on the screen for 250 ms, requiring participants to estimate the number of dots displayed. They were asked to estimate a total of eight images and input their responses after each presentation. Following the estimation phase, participants received an instruction indicating that their overestimation bias had led to their assignment to the “overestimation group”.

Participants were randomly assigned to either an in-group or an out-group condition. In the in-group condition, individuals were notified that they would be paired with members of the same group for the subsequent trust game, indicating that both the participant and their opponent belonged to the “overestimation group”. Conversely, in the out-group condition, participants were informed of their pairing with members from a different group, signifying that their opponent was from the “underestimation group”. Each participant engaged in seven sequential rounds of the trust game with their assigned partner. The initial four rounds constituted the trust-building phase, wherein the counterpart returned half of any amount invested by the participant. The subsequent rounds, specifically Round 5 and 6, represented the trust violation phase, characterized by an absence of return. Upon the termination of the trust game in Round 6, the game was interrupted by an apology from the partner: “I apologize for breaching your trust; I am remorseful and pledge to rectify my actions in the subsequent round”. Round 7 initiated the trust-rebuilding phase, where the partner returned half of the received amount. Post-completion of the seventh round, participants were instructed to complete the Trust Propensity Scale and the Trait Forgiveness Scale.

### 2.2. Results

#### 2.2.1. Manipulation Checks of Trust Violation

This study initially assessed the efficacy of trust violation manipulation. A repeated measures analysis of variance (ANOVA) was executed to discern differences in the trust game between in-group and out-group participants during Rounds 5 and 6, with gender, age, trust propensity, and forgiveness traits as covariates. The analysis revealed a significant main effect for the rounds, *F* = 11.06, *p* = 0.001, partial *η*^2^ = 0.07, indicating that the investment in Round 5 (64.28 ± 27.36) was notably higher than in Round 6 (45.38 ± 26.22). However, no significant main effect for the group was observed (*F* = 0.35, *p* = 0.56, partial *η*^2^ = 0.000), nor was there a significant interaction between the group and round (*F* = 0.10, *p* = 0.75, partial *η*^2^ = 0.000).

#### 2.2.2. The Influence of In- and Out-Groups on Trust Repair

**Figure 1:**
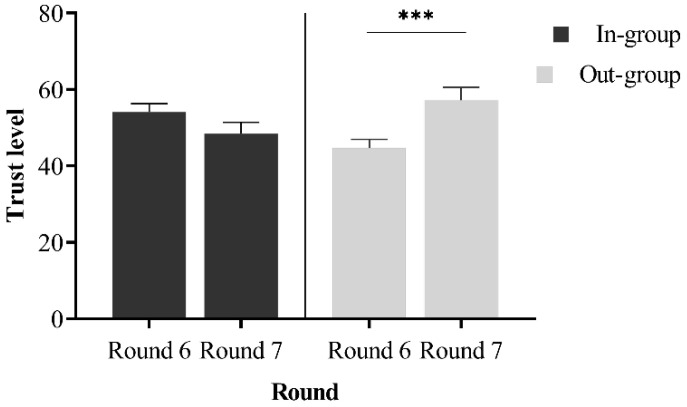
Trust repair differences between in- and out-groups. Asterisks indicate statistically significant differences between conditions (*** p < 0.001). The error line represents standard error.

In the trust game, the seventh round served as a phase for trust restoration. Following the sixth round and before the seventh, an apology session was implemented to aid in rebuilding trust. A repeated measures ANOVA was conducted to analyze the differences in investment between in-group and out-group participants in the sixth and seventh rounds, with gender, age, trust propensity, and forgiveness traits as control variables. The results (see Figure 1) revealed a significant interaction effect, *F* = 5.05, *p* = 0.026, partial *η^2^* = 0.03. Notably, there was no significant difference in investment between in-group members in Round 6 (46.12 ± 24.60) and Round 7 (48.43 ± 26.28). Conversely, out-group participants demonstrated a significant difference, with higher investment in Round 7 (57.17 ± 28.89) compared to Round 6 (44.60 ± 27.97). No other main effects were significant (*p* > 0.05). This finding suggests that individuals exhibit a “black sheep effect” in intergroup trust recovery, showing greater tolerance towards out-group members who admit their mistakes, making it easier to restore trust.

## 3. Study 2

Study 2 aimed to investigate the influence of group identities on intergroup trust repair under different situations (competition vs. cooperation). A 2 × 2 between-subjects design was employed, with group identity (in-group vs. out-group) and interaction context (competition vs. cooperation) as independent variables. Participants were assigned to in-group and out-group categories through a point estimation task in the minimal group paradigm, with the study focusing on the effects of these identities on trust repair across different interaction situations. We hypothesized that in competitive situations, individuals will still exhibit the “black sheep effect” in the process of group trust repair. In contrast, in cooperative situations, individuals are capable of effectively restoring trust with both in-groups and out-groups.

### 3.1. Method

#### 3.1.1. Participant

A sample of 161 nonpsychology undergraduate students from a priori power analyses (*α* = 0.05, *power* = 0.80) were recruited, consisting of 79 males and 82 females, with an average age of 20.80 years (*SD* = 2.17). Eligible participants had normal or corrected vision, were right-handed, and provided voluntary consent for the study. Following the experiment, participants received compensation proportional to their performance.

#### 3.1.2. Measures and Procedure

The trust game, the Trust Propensity Scale, and Trait Forgiveness Scale were the same as in Study 1.

The procedure for Study 2 closely resembled that of Study 1. Specifically, after participants were informed of their allocation to the “overestimation group”, they received distinct situational instructions designed to foster either competitive or cooperative scenarios. In the competitive condition, participants were informed of an impending competition, irrespective of whether their opponent in the subsequent task was a group member. The individual amassing the most tokens would earn an additional reward. In contrast, the cooperative condition communicated a collaborative relationship, with the collective acquisition of tokens directly influencing the magnitude of the participants’ ultimate reward. Subsequently, participants were randomly assigned to either an in-group or an out-group and proceeded to engage in a trust game across seven rounds, mirroring the structure of Study 1.

### 3.2. Result

A repeated measures ANOVA was employed to assess the impact of trust violation manipulation. The analysis revealed a significant main effect of rounds, *F* = 4.16, *p* = 0.042, partial *η*^2^ = 0.03, with the investment in Round 5 (71.43 ± 29.68) significantly higher than that in Round 6 (41.41 ± 28.67). Additionally, there was a marginally significant interaction between context and round, *F* = 3.55, *p* = 0.062, partial *η*² = 0.02. In Round 5, investments were higher in the cooperative (78.52 ± 26.58) compared to the competitive situation (64.52 ± 35.88), but this difference was not significant in Round 6 (cooperative: 44.82 ± 29.16; competitive: 38.07 ± 27.96). No other main effects or interactions reached statistical significance (*p* > 0.05).

Then, the study employed a repeated measures analysis of variance (ANOVA) to examine the impact of interactive situations and group identity on the process of intergroup trust repair. The analysis revealed a significant interaction between interaction context and round, *F* = 5.12, *p* = 0.025, partial *η*^2^ = 0.03. Specifically, within the cooperative situation, the mean investment in Round 7 (61.71 ± 31.46) was significantly greater than that in Round 6 (44.82 ± 29.16). Conversely, in the competitive situation, there was no significant difference in the mean investment between the two rounds (Round 6: 38.07 ± 27.96; Round 7: 45.14 ± 35.22). Furthermore, the interaction between group identity and round, *F* = 5.54, *p* = 0.020, partial *η^2^* = 0.04, indicated a significant difference in investment amounts for out-group members, with higher investments in Round 7 compared to Round 6. In contrast, for in-group participants, there was no significant difference in investments between Rounds 6 and 7.

**Figure 2:**
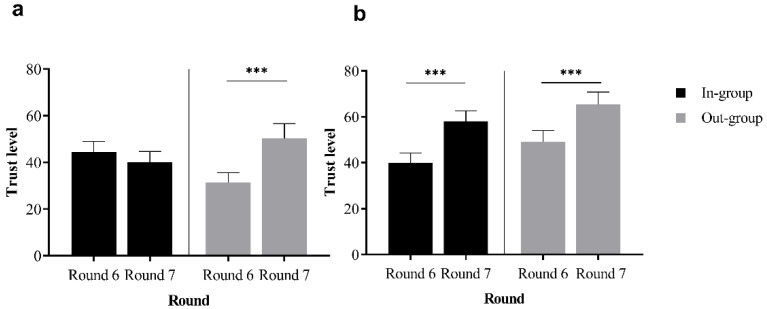
Intergroup trust repair in cooperative and competitive situations. (**a**) The competitive situations. (**b**) The cooperative situations. Asterisks indicate statistically significant differences between conditions (*** p < 0.001). The error line represents standard error.

Importantly, a significant interaction was observed between interaction context, group identity, and rounds (*F* = 3.97, *p* = 0.048, partial *η^2^* = 0.03, see Figure 2). In the cooperative situation, both the in-group (Round 6: 40.93 ± 27.09; Round 7: 58.32 ± 29.56) and the out-group (Round 6: 49.03 ± 31.06; Round 7: 65.37 ± 33.41) displayed a notable increase in investment from Round 6 to Round 7. However, in the competitive situation, the out-group exhibited a significant difference, with higher investment in Round 7 (50.38 ± 39.67) compared to Round 6 (31.42 ± 22.64), while the in-group did not show any significant change between the two rounds (Round 6: 44.56 ± 28.19; Round 7: 40.02 ± 29.88). This finding underscores the “black sheep effect” of intergroup trust repair, which is more pronounced for out-group members in competitive situations.

## 4. Discussion

### 4.1. The “Black Sheep Effect” in Intergroup Repair Trust

In relatively unfamiliar group interaction settings, the delineation of in-group and out-group classifications enables individuals to rapidly form fundamental perceptions and attitudes toward their interaction partners, thus shaping interpersonal behaviors [37]. The present study concentrates on the dynamics of trust repair and examines the influence of group identity on the efficacy of trust restoration efforts. The research revealed that individuals are inclined to prioritize trust repair with out-group members over those within their in-group, leading to a more pronounced effect when repairing trust with the out-group—a phenomenon referred to as the “black sheep effect”. These findings are consistent with prior studies, including one by Wu and Gao (2018) [17], which observed that individuals are more critical and less inclined to re-establish trust with in-group deviants compared to out-group members.

The norm focus theory posits that the “black sheep effect” is predominantly driven by two factors: the violation of expectations and the desire to preserve group norms [21]. Expectation violation entails an individual’s cognitive assessment of the divergence between anticipated outcomes and actual events. Given the shared values within in-groups, members anticipate greater levels of cooperation and trustworthiness from their peers. The deviation of in-group members from these expectations triggers more intense negative emotions, such as anger and disgust, often culminating in stricter punishment [38,39]. This study revealed that individuals anticipate higher trustworthiness and reciprocity from in-group members. However, when a member betrays this trust by failing to fulfill these expectations, it prompts the individual to enforce punishment, thereby diminishing the efficacy of trust repair and manifesting the “black sheep effect”. Additionally, to maintain group norms, individuals are inclined to enforce stringent sanctions on those within the group who deviate from these norms [40,41]. Group norms are the behavioral standards internalized and recognized by group members, which are instrumental for the group’s formation, functioning, and sustenance. In this study, a “mutually beneficial” norm was established through initial trust-building interactions. When this norm was subsequently breached, the act of upholding the group norm involved punishing the in-group member responsible for the breach, thereby eliciting the “black sheep effect”.

### 4.2. The Moderating Effects of Cooperation and Competition on Trust Repair in In- and Out-Groups

Study 2 aimed to investigate the moderating influence of group interaction dynamics (cooperation vs. competition) on intergroup trust repair. The results demonstrated that in the cooperative situation, both in-group and out-group trust were effectively restored. Conversely, in the competitive situation, trust repair was more pronounced among out-group members compared to in-group, revealing a “black sheep effect”. Research supports that cooperation fosters trust and prosocial behavior; for instance, after cooperative games, children exhibit reduced aggression and increased altruism [42]. Cooperative environments facilitate reciprocity due to shared goals and reduced perceived threats [43], enabling individuals to rebuild trust for mutual benefit. However, competition accentuates group differences, potentially heightening the perceived threat of deviant behavior to in-group identity. This triggers defensive responses, resulting in heightened negative emotions and stricter punishment towards in-group members [44,45]. Consequently, when in-group members display deviant behavior, the desire to maintain a positive group image and identity may lead to harsher punishment, exemplifying the “black sheep effect”.

### 4.3. Implications and Limitations

The study at hand delves into the pivotal role of intergroup interaction in social contexts, highlighting the profound impact of group identity on individual behaviors and decision-making processes. By examining the contrasting responses to rule-breaking within groups, namely, in-group favoritism and the black sheep effect, the research sheds light on the complex dynamics of trust and its restoration in the context of intergroup relations. The theoretical significance lies in elucidating the nuanced influence of group identity on trust dynamics and the moderating role of interaction types, such as cooperation and competition, in trust restoration processes. Practically, these insights contribute to enhancing team collaboration efficiency by offering a framework for understanding and addressing trust issues within diverse groups, thereby facilitating conflict mitigation and fostering a harmonious intergroup environment. Moreover, the findings provide a foundation for policymakers and practitioners to design interventions aimed at building and repairing trust, which is particularly relevant in the management of diverse teams in a globalized context. The implications extend to cross-cultural understanding, suggesting that the study’s findings could inform trust and group interaction research across different cultural settings, promoting effective cooperation and mutual understanding beyond the specific conditions examined. 

However, the study is not without limitations. It utilized a minimal group paradigm, employing point-estimation tasks to manipulate group membership. While this method effectively controls extraneous variables, it may not accurately represent real-life group dynamics, as participants did not interact with either in-group or out-group members and remained anonymous to each other [46], thus partially compromising ecological validity. Future research could improve ecological validity by incorporating natural and social cues, such as gender, ethnicity, peers, and schools, thereby reinforcing the stability of the findings. Additionally, the reliance on cross-sectional or experimental designs may not fully capture the dynamic nature of trust development over time. Longitudinal studies could offer a more in-depth understanding of trust evolution in response to varying interaction patterns. Finally, while the “black sheep effect” observed in competitive situations is noteworthy, further empirical evidence is necessary to ascertain its prevalence and to investigate potential mitigating factors. Additionally, future research could incorporate cross-cultural studies to further examine the applicability and differences of these results in various cultural contexts, highlighting the connection between sociocultural factors and individual behavior.

## 5. Conclusions

The study investigated the influence of group identity on intergroup trust repair, with a particular focus on the moderating effect of interaction contexts (cooperation versus competition). The findings uncovered a distinctive pattern, referred to as the “black sheep effect”, where individuals demonstrated enhanced trust repair with out-group members compared to their in-group counterparts. Notably, this effect was predominantly observed in competitive situations, while trust repair exhibited equal efficacy among in-group and out-group members in cooperative situations.

## Data Availability

The data that support the findings of this study are available from the corresponding author upon reasonable request.
